# Sclerosing Peritonitis: A Rare but Fatal Complication of Peritoneal Inflammation

**DOI:** 10.1155/2012/709673

**Published:** 2012-03-26

**Authors:** Monika Merkle, Markus Wörnle

**Affiliations:** Medizinische Poliklinik, Campus Innenstadt, Klinikum der LMU, Pettenkoferstrasse 8a, 80336 München, Germany

## Abstract

Sclerosing peritonitis is a rare form of peritoneal inflammation with an often fatal outcome. The major risk factor of sclerosing peritonitis is peritoneal dialysis treatment but it can also occur following renal or liver transplantation or be associated with certain drug treatment. This article gives an overview of reasons and treatment options for sclerosing peritonitis and shows a summery of current literature about sclerosing peritonitis.

## 

Sclerosing peritonitis (SP) is a rare form of peritoneal inflammation which involves both the visceral and the parietal surfaces of the abdominal cavity. SP is characterized by a fibrous thickening of the peritoneum and is reported to complicate peritoneal dialysis, certain drugs, and infectious peritonitis. SP was first described in 1907 [1], with the term “abdominal cocoon” being in use since 1978 [2]. 

Due to the low incidence and the relatively slow disease progress, there is no valuable data with respect to the comparative incidence of SP related to dialysis versus SP without any known risk factor [3, 4]. Published data indicate prevalence rates between 0.54% and 0.9% [5–7]. The etiology of SP remains unknown and presumably is multifactorial [3, 4]. There is no distinct relationship between the renal disease leading to end stage renal failure and the development of SP.

The major risk factor for SP is peritoneal dialysis treatment (PD) [8, 9]. Indeed, the utilization of the peritoneal cavity for dialysis therapy is associated with an increased risk of structural and functional damage to the peritoneal membrane. PD-related risk factors such as duration of therapy, poor biocompatibility of dialysis solutions, and peritonitis are considered to be important for the development of SP, with the duration of PD being the most relevant single factor. SP usually occurs in patients receiving PD for more than 4 or 5 years and the incidence of SP in patients on PD treatment for less than 2 years is very low [6, 7]. Nonphysiologic dialysis solutions may induce a chronic sterile inflammation in the peritoneal cavity with upregulation of several cytokines resulting in collagen synthesis by mesothelial cells and fibroblasts. Moreover, the high concentrations of glucose and lactate as much as the low pH of the dialysis solutions and bioincompatible substances directly damage the peritoneal membrane. Alike bacterial or fungal peritonitis, they lead to the loss of mesothelium and a decline in fibrinolytic capacity of the peritoneal membrane, and might thereby contribute to the development of SP [7]. The largest observational study published by Kim et al. [6] included 4.290 PD patients followed from 1981 to 2002, 34 of whom developed SP, corresponding to an overall prevalence of 0.79%. The male to female ratio in these patients was 1 : 1, their median age was 44.5 years (range 19–66 years) and the median duration of PD treatment until the diagnosis of SP was 64 months (range 9–144 months), with 23 of 34 patients (68%) having been on PD for more than 4 years. Remarkably, 27 of the 34 SP patients (79%) had a medical history of peritonitis, including two cases of fungal peritonitis, with a median of 6 and at most 15 episodes of PD-related peritonitis ultimately leading to catheter removal. 18 patients were diagnosed by clinical and radiological methods, the remaining 16 were diagnosed surgically. 11 patients were treated by laparotomy with excision of the sclerosed peritoneum. The overall mortality in SP patients was 24%, the mortality in patients who had undergone surgical treatment was 43%.

Betablockers are also supposed to contribute to the development of SP [9], which has most commonly described in association with practolol, but also metoprolol, propranolol and atenolol [10, 11]. The pathomechanism is not entirely clear, but probably relates to the inhibition of surfactant release by betablockers [9]. Clinical studies found a history of corresponding medication in many patients with SP, yet the factual significance of this drug class for the development of SP could not be clarified [6]. Antibiotics used for treatment of peritonitis are as well discussed to be a risk factor for SP [12].

Clinical signs of patients developing SP while on peritoneal dialysis therapy are ultrafiltration and clearance failure. Furthermore, small bowel obstruction due to encapsulation, adhesions, and mural fibrosis with anorexia, nausea, and vomiting is frequently observed and ultimately entails malnutrition (Table 1) [9]. Following termination of PD treatment, patients with SP often develop ascites [13]. Histopathologic examination reveals fibrosis of the entire peritoneum with substantial thickening. This differs from simple sclerosis, which is also found in patients with ascites or peritoneal dialysis and is usually not associated with a clinically relevant disease progress [14].

There are some reports of SP as late sequelae of PD, typically occurring in patients who had previously undergone successful renal transplantation [15]. Adamidis et al. reported a case of a former PD patient who presented 2 years after renal transplantation with abdominal discomfort, vomiting, and malnutrition due to SP. Despite the initial conservative treatment, for persistence of symptoms he underwent surgical treatment later on and recovered without any further complication [16]. Bowers et al. reported 3 cases of SP in former, meanwhile successfully transplanted PD patients. Each of these patients suffered from a mechanical small bowel obstruction secondary to a densely fibrosing and encasing peel of reactive tissue visibly different from the usual postoperative adhesions [17]. Morrow et al. reported the case of a 55-year-old woman with end-stage renal disease secondary to systemic lupus erythematodes who had received two renal transplants within 15 years; between the two transplantations she had been on PD for 5 years. Following her second successful kidney transplant, she presented with persistent nausea and vomiting and was diagnosed to have SP by CT scan. Due to failure of conservative management she underwent exploratory laparotomy with extensive lysis of adhesions and the postoperative course was complicated by intolerance to feedings. 3 months later, a second laparotomy had to be performed due to obstruction of the proximal jejunum. Intraoperative findings were similar to the prior ones. Extensive lysis of adhesions and Billroth II gastrojejunostomy bypass is done, and the patient recovered tolerating a regular diet [15].  Table 2 summarizes the publications relating to patients with SP following renal transplantation, the respective therapy, and patient survival. From the sporadic case reports, however, no preferential association with determinate immunosuppressive regimens could be inferred nor be corroborated by any other evidence. Anyway, even though being extremely rare, SP has to be included in the differential diagnosis of every renal transplant patient with prior PD treatment who presents with unexplained malnutrition and symptoms of abdominal obstruction.

SP has been also described in association with other diseases and as an idiopathic form [18]. The idiopathic form predominantly occurs in adolescent females and a possible relationship to retrograde menstruation has been discussed [19, 20]. SP has also been reported in patients with ventriculoperitoneal shunts [21]. Moreover, patients with end-stage liver disease waiting for liver transplantation are at a higher risk for developing SP. With continuous peritoneal irritation from ascites and recurrent spontaneous bacterial peritonitis, these patients suffer from two independent conditions identified as risk factors for SP. SP has also been reported in patients after liver transplantation. The typical symptoms are abdominal pain, refractory ascites, bowel obstruction, and malnutrition [22–24]. In a large prospective study including 1.800 liver transplant recipients, Maguire et al. reported on 5 patients aged 16 to 57 years who developed SP after liver transplantation. None of these patients had a peritoneal-venous shunt or had undergone peritoneal dialysis. All 5 patients developed fever as early as 66 ± 21 hours posttransplant, with confirmation of bacterial peritonitis in two patients and additional symptoms including epigastric discomfort and intermittent vomiting occurring 12 ± 10 days later. While abdominal CT consistently showed marked ascites confined to definite areas of the abdomen, ultrasound and intestinal contrast studies were not diagnostic. All patients underwent an early second laparotomy with removal of an abdominal cocoon membrane. 4 of 5 patients survived without long-term sequelae [22]. Mekeel et al. reported on 3 patients suffering from SP after liver transplantation, namely, two 42- and 62-year-old males with end-stage liver disease due to hepatitis C infection without significant other past medical or surgical history and a 59-year-old alcoholic. All 3 patients had massive refractory ascites with episodes of spontaneous bacterial peritonitis prior to transplantation. Two patients had evidence of a fibrous peel already at the time of transplantation. Postoperatively, all 3 patients continued to have refractory ascites and episodes of peritonitis, along with partial small bowel obstructions, abdominal pain, and malnutrition. Beyond that, in two patients a constriction of the graft, including biliary as well as inferior vena cava and outflow obstruction occurred [23]. Lin et al. reported on a patient with hepatitis-B-related hepatocellular carcinoma and a prior peritoneal-venous shunt who developed SP with small bowel obstruction two weeks after liver transplantation [24]. Accordingly, not only SP apparently occurs rather early in recipients of liver transplants, but its diagnosis proves to be difficult.  Table 3 summarizes the publications relating to patients with SP following liver transplantation, the respective therapy, and patient survival.

 At last, SP has been observed in children who had previously undergone intestinal transplantation; it is characterized by progressive serositis as well as fibrous changes of the intestinal allograft mesentery and serosa with progressive contraction culminating in intestinal obstruction. The native intestinal tract is always spared. The causes of SP associated with intestinal transplantation are not completely understood. No distinct risk factors as cold ischemia time or severity of rejection could be identified. In an adult patient, sclerosing mesenteritis ascribed to a vascular form of antibody-mediated rejection has been reported after a combined liver-intestine transplantation; therefore, chronic rejection may be considered as a trigger for the development of SP in intestinal transplant recipients. Macedo et al. retrospectively reviewed the medical records of 121 children who underwent intestinal transplantation between 1990 and 2003; in this cohort, three children (2.4%) suffered from distal ileal obstruction of the allograft intestine secondary to SP. The indication for their intestinal transplantation was intestinal failure secondary to gastroschisis in two patients and midgut volvulus in one patient. All patients had become independent of total parenteral nutrition and were asymptomatic until the diagnosis of SP. The mean time to presentation with symptoms of SP was 6.6 years (range 5.3–8 years) from intestinal transplantation. Laparotomy was performed in all three patients and showed serositis, dense fibrous adhesions, and a contracted mesentery. In all patients adhesiolysis and segmental resection of the distal ileum were performed. Two patients died 2.5 and 3, respectively, years after transplantation [25].

 The diagnosis of SP is made clinically, radiographically, or by laparotomy. Characteristic radiographic findings can be visualized on small bowel follow-through series or CT imaging. CT scans typically show small bowel congregated in the center of the abdomen, thickened peritoneum, or large locular fluid collections. In addition, a delayed transit of contrast can be observed on fluoroscopy [15, 26–28].

There is no expert agreement on whether the treatment of choice should be surgical or conservative. As obvious from the low incidence of SP, there are no respective clinical studies comparing the different therapeutical approaches, and experience is limited to case reports. Treatment of PD patients with SP includes cessation of PD therapy and conversion to hemodialysis. Bowel rest and total parenteral nutrition seem to alleviate symptoms substantially. Medical treatment regimens based on corticosteroids and methotrexate have been recommended [29, 30]; for its known effects on other fibrotic diseases, including retroperitoneal fibrosis and desmoid tumors, tamoxifen has been adopted as therapeutic agent in SP as well [31]. However, there are minimal data supporting a positive effect of any of the cited medical treatment options. Surgical treatment is exclusively recommended for patients suffering from intestinal obstruction [6]. The mortality of SP has been reported as high as 24% and may attain 60% in patients who is managed operatively [6] due to perioperative complications like anastomosis insufficiency, intraabdominal infections, and enterocutaneous fistulas.

In conclusion, SP is a rare but serious complication affecting mainly patients on PD treatment. SP is a potentially late sequela of PD and can be found as well in patients after liver or intestinal transplantation without a history of PD; hence, it should be included in the differential diagnosis of every case of unexplained malnutrition and abdominal obstruction, especially in patients after solid organ transplantation.

## Figures and Tables

**Table 1:** Clinical characteristic of sclerosing peritonitis (SP).

Abdominal pain	
Nausea	
Vomiting	
Weight loss	
Loss of ultrafiltraion (in PD patients)	
Blood-stained dialysate (in PD patients)	

**Table 2:** Sclerosing peritonitis after kidney transplantation.

	No. of cases	Therapy	Survival : death
Bowers et al.	3	Surgery	3 : 0
Clin transplant 1994			
Morrow et al.	1	Surgery	1 : 0
Dig Dis Sci 2011			
Adamidis et al.	1	Surgery	1 : 0
Ren Fail 2011			

**Table 3:** Sclerosing peritonitis after liver transplantation.

	No. of cases	Therapy	Survival : death
Maguire et al.	5	Surgery	4 : 1
American Journal of Surgery 2001			
Lin et al.	1	Surgery	1 : 0
World Journal Gastroenterol 2005			
Mekeel et al.	3	Surgery	0 : 3
Liver Transplantation 2009			
